# Inhibitory Effects of Standardized Extracts of *Phyllanthus amarus* and *Phyllanthus urinaria* and Their Marker Compounds on Phagocytic Activity of Human Neutrophils

**DOI:** 10.1155/2013/603634

**Published:** 2013-05-02

**Authors:** Yuandani, Menaga Ilangkovan, Ibrahim Jantan, Hazni Falina Mohamad, Khairana Husain, Amirul Faiz Abdul Razak

**Affiliations:** Drug and Herbal Research Center, Faculty of Pharmacy, Universiti Kebangsaan Malaysia, Jalan Raja Muda Abdul Aziz, Kuala Lumpur 50300, Malaysia

## Abstract

The standardized methanol extracts of *Phyllanthus amarus* and *P. urinaria*, collected from Malaysia and Indonesia, and their isolated chemical markers, phyllanthin and hypophyllanthin, were evaluated for their effects on the chemotaxis, phagocytosis and chemiluminescence of human phagocytes. All the plant extracts strongly inhibited the migration of polymorphonuclear leukocytes (PMNs) with the Malaysian *P. amarus* showing the strongest inhibitory activity (IC_50_ value, 1.1 **µ**g/mL). There was moderate inhibition by the extracts of the bacteria engulfment by the phagocytes with the Malaysian *P. amarus* exhibiting the highest inhibition (50.8% of phagocytizing cells). The Malaysian *P. amarus* and *P. urinaria* showed strong reactive oxygen species (ROS) inhibitory activity, with both extracts exhibiting IC_50_ value of 0.7 **µ**g/mL. Phyllanthin and hypophyllanthin exhibited relatively strong activity against PMNs chemotaxis, with IC_50_ values slightly lower than that of ibuprofen (1.4 **µ**g/mL). Phyllanthin exhibited strong inhibitory activity on the oxidative burst with an IC_50_ value comparable to that of aspirin (1.9 **µ**g/mL). Phyllanthin exhibited strong engulfment inhibitory activity with percentage of phagocytizing cells of 14.2 and 27.1% for neutrophils and monocytes, respectively. The strong inhibitory activity of the extracts was due to the presence of high amounts of phyllanthin and hypophyllanthin although other constituents may also contribute.

## 1. Introduction

Phagocytosis is an important response of innate immunity which is mediated by professional phagocytes such as polymorphonuclear neutrophils (PMNs), peripheral blood mononuclear, and macrophage cells [1, 2]. Phagocytes play important roles in our innate immune defence against infectious microbes and in activating the adaptive immune response [3]. Phagocytic activity consists of several steps, that is, migration of phagocyte cells to the site of infection, adherence to vascular endothelial cells, recognition of the target structures, and subsequent engulfment of the pathogen, followed by intracellular destruction [4].

 At the earliest stage of immune response, active recruitment of neutrophils to sites of infection is fundamentally important. This process involves mobilizations of PMNs from circulation in response to host- and pathogen-derived chemotactic factors. Phagocytes pass through the capillary wall, surveying tissue, membranes, and lymphatic organs for signs of tissue distress and the presence of chemoattractant [4]. Endogenous substances such as interleukin 8 (IL-8), leukotriene B4 (LTB4), platelet-activating factor (PAF), and exogenous substances such as formyl methionyl-leucyl-phenylalanine (fMLP) derived from bacterial cell products are the important neutrophil chemoattractants [5]. The phagocytes adhere stably to the endothelium cells because they possess cell surface expression of all three CD11/CD18 leucocyte integrins [6]. The phagocytes directly recognize surface bound or freely secreted molecules produced by pathogen and interact directly with a number of pattern-recognition receptor expressed on the cell surface, such as TLRs, CD14, and Fc*γ*R [5, 7]. After adherence of the pathogen to the surface of phagocytes through recognition receptor, engulfment phase is initiated. The pathogens are then destroyed by microbicidal mechanism that is often referred to as oxidative burst [5]. 

 The oxidative burst involves the generation of superoxides by NADPH-oxidase complex through a series of molecular reaction that consume oxygen. Myeloperoxidase (MPO) in the phagosome catalyzes the transformation of superoxide into a variety of toxic molecules for microorganisms, such as hypochlorous acid, chlorines, chloramines, hydroxyl radicals, and single oxygen [3]. Besides the defensive roles during the infections, the phagocyte-microbe interactions when excessively or inappropriately deployed can damage host tissues and contribute to the pathogeny of various immune and nonimmune chronic inflammatory diseases, including some rheumatoid disorders. Therefore, the inhibitors of phagocyte reactive oxygen species (ROS) production can be used in the treatment of a variety of disorders including inflammation [8].

 Many therapeutic effects of plant extracts have been suggested to be due to their influence on the immune system of the human body [9]. Many herbal preparations such as *Tinospora cordifolia, Centella asiatica, Phyllanthus debilis, Trigonella foenum graecum, Pouteria cambodiana, Panax ginseng*, and *Picrorhiza scrophulariiflora* have been shown to alter the immune function and possess a wide array of immunomodulatory effects [10–13]. Our previous study on the screening of 20 medicinal plants for their phagocytic properties has indicated that the methanol extracts of some plants including *Phyllanthus amarus* exhibited strong immunomodulatory effects on polymorphonuclear neutrophils and macrophage cells [14]. *P. amarus* Schum. & Thonn. and *P. urinaria* Linn (family Euphorbiaceae) are widely used in traditional medicine to treat various diseases such as viral hepatitis, diarrhoea, jaundice kidney disorders, influenza, diabetes, bronchial infections, sores, swelling, itchiness, cardiovascular problems, and inflammatory disorders [15, 16]. 

*P. amarus* was found to be rich in lignans, polyphenols, flavonoids, hydrolysable tannins, triterpenes, sterols, and alkaloids [15–18]. The extract and compounds isolated from *P. amarus* have shown a wide spectrum of pharmacological activities including antiviral, hepatoprotective anti-inflammatory, antioxidant, antiplasmodial, antimalarial, antidiabetic, hypolipidemic, antihyperuremic, nephroprotective, and diuretic properties [18–24]. The extract and purified lignans such as phyltetralin, nirtetralin, and niranthin from *P. amarus* exhibited important *in vivo* and *in vitro* anti-inflammatory actions [20]. Several studies have indicated that *P. amarus* was able to suppress the growth and replication of hepatitis B virus [23]. The hepatoprotective effect of *P. amarus* and its ability to protect hepatocytes against carbon tetrachloride, paracetamol, ethanol, aflatoxin B1, and galactosamine-induced liver toxicity in various animal models have been well documented [24]. Phyllanthin and hypophyllanthin present in *P. amarus* have been shown to inactivate hepatitis B, both *in vitro* and *in vivo*, and these two lignans were also reported to be the active principles accountable for the hepatoprotective property of many *Phyllanthus* species [24]. Phytochemical studies on *P. urinaria* have resulted in the isolation of various compounds, mainly lignans, flavonoids, tannins, and other benzenoid constituents [25–27]. Various biological activities of *P. urinaria* have been reported, including hepatoprotective effect, antihepatitis B virus, anti-Epstein-Barr virus, antiretroviral reverse transcriptase, and antiherpes simplex virus type I and type 2 [28, 29]. Antioxidant and inflammatory mediator's growth inhibitory effects of compounds isolated from *P. urinaria* have been reported [30]. 

 In the present study the standardized methanol extracts of *P. amarus* and *P. urinaria*, collected from Malaysia and Indonesia, were investigated for their inhibitory effects on phagocytic activity of human neutrophils. The biochemical markers of the extracts were isolated, and their immunomodulatory effects were determined in an effort to correlate the inhibitory activity of the extracts with their active components. The results of this study may provide some insights on the ability of these plants and their marker compounds to modulate the innate immune response of phagocytes at different steps.

## 2. Materials and Methods

### 2.1. Chemicals and Reagents

 The chemicals used in this study were of analytical grade. Serum opsonized zymosan A (*Saccharomyces cerevisiae* suspensions and serum), luminol (3-aminophthalhydrazide), phosphate buffer saline tablet (PBS), Hanks Balance Salt Solutions (HBSS), Ficoll, Hanks Balance Salt Solution (HBSS), N-formyl-methionylleucyl-phenylalanine (fMLP), acetyl salicylic acid (purity 99%), ibuprofen (purity 99%), and dimethylsulfoxide (DMSO) were purchased from Sigma (St Louis, MO, USA). Fetal calf serum was obtained from PAA Laboratories (USA). Chemiluminescence measurements were carried out on a Luminoskan Ascent luminometer (Thermo Scientific, UK). fMLP was stored as a stock solution of 10^−8^ M in DMSO at −80°C and diluted in Hanks solution, prior to assay. Haematoxylin and xylene for staining were obtained from BDH, UK. A Boyden 48-well chamber with a 2 *μ*m polycarbonate membrane filter separating the upper and lower compartments was purchased from Neuro Probe (Cabin John, MD, USA). Phyllanthin and hypophyllanthin standards (purity >98%) were purchased from ChromaDex (CA, USA). Methanol HPLC grade, acetonitrile HPLC grade, and, trifluoroacetic acid AR grade were obtained from E-Merck. Phagotest kit was obtained from Glycotope Technology, Germany. The flow cytometer BDFACS Canto II equipped with 488 nm argon-ion laser was used. A CO_2_ incubator (Shell Lab, USA), light microscope, and high-performance liquid chromatograph (Waters 2998) (Leitz Watzler, Germany) were also used in this assay. Molecular weights of the compounds were recorded by ESIMS using ESI-TOFF MS (Bruker MicroToF-Q 86, Switzerland). The ^1^H and ^13^C NMR spectra were carried out on a JOEL NMR 500 MHz (JOEL Ltd, Japan) with TMS as internal standard.

### 2.2. Plant Collection

 The whole plants of *Phyllanthus amarus* and *P. urinaria* were collected from Marang, Kuala Terengganu, Malaysia and Tanjung Anom, Northern Sumatera, Indonesia, between February and June 2012. The voucher specimens (*P. amarus* UKMB 30078 and *P. urinaria* UKMB 30077) were identified by Dr Abdul Latif Mohamad of Faculty of Science and Technology, Universiti Kebangsaan Malaysia (UKM), and deposited at the Herbarium of UKM, Bangi, Malaysia.

### 2.3. Extraction and Isolation of Phyllanthin and Hypophyllanthin

 The plant materials were allowed to dry under shade. 500 g of dried material of each plant sample were ground and macerated in methanol at the ratio of 1 : 10 (w/v). The extraction was repeated thrice on the residue. The filtrates were combined and the solvent was removed under reduced pressure to obtain extracts of *P. amarus* (Malaysia, 55.2 g, 11.04% w/w; Indonesia, 49.7 g, 10.18% w/w) and *P. urinaria* (Malaysia, 52.7 g, 10.54% w/w; Indonesia, 47.2 g, 9.44% w/w). Ten g of *P. amarus* extract was fractionated by vacuum liquid chromatography (VLC) on silica gel type H (10–40 *μ*m, 7 × 30 cm) and eluted with a gradient system of hexane :  CHCl_3_ (10 : 0–1 : 9, v/v) and CHCl_3_: MeOH (10 : 0–0 : 10, v/v); repeated silica gel column (40–63 *μ*m, 3 × 60 cm) was eluted with a gradient system of n-hexane : ethyl acetate (10 : 0–1 : 9, v/v), followed by recrystallization from EtOAc: hexane to yield different amounts of phyllanthin (Malaysian sample: 228.5 mg, 2.29%; Indonesian sample: 115.0 mg, 1.15%) and hypophyllanthin (Malaysian sample: 321.2 mg, 3.21%; Indonesian sample: 235.5 mg, 2.35%). Purity of the compounds was >98%, based on their physicochemical properties, NMR and ESI-MS data.

### 2.4. Standardization of the Methanol Extracts of Phyllanthus Species by HPLC

 Twenty mg of the methanol extracts of the plant in 10 mL of methanol and 1 mg each of the reference standards of phyllanthin and hypophyllanthin in 1 mL of methanol were filtered through 0.45 *μ*m Millipore Millex PTFE membrane (Maidstone, Kent, UK) before injection. The diluted solutions of the extracts and the reference standards were analyzed separately by HPLC using the following conditions: column: Reverse Phase, C-18 column (250 mm × 4.6 mm i.d., 5 *μ*m, Xbridge, Waters, Ireland), detector: PDA (Waters 2998), wavelength: 205 nm, flow rate: 0.4 mL/min, Mobile phase: A. acetonitrile : B. water (acidified with 0.1% orthophosphoric acid) isocratically eluted with 5% B, and then increased to 95% over 20 min and had been hold at 95% for 15 min. 

### 2.5. Validation Procedures for HPLC Analysis

 The reversed-phase HPLC method was validated by determination of linearity, precision, limits of quantification (LOQ), and detection (LOD). Linearity was evaluated by linear calibration analysis and correlation coefficient (R2), calculated from the calibration curves. Calibration standards were prepared at concentrations of 250, 125, 62.5, and 31.25 *μ*g/mL of phyllanthin and hypophyllanthin. A graph was plotted for the area versus concentration of corresponding compound. Precision of the method was determined by intra-assay and interassay validation. Separately, one concentration of extract (2 mg/mL) and reference compounds (1 mg/mL) were injected three times in one day and on three different days. LOD and LOQ were calculated from the RSD and slope (*S*) of the calibration curves using equations: LOD = 3.3 × (RSD/*S*) and LOQ = 10 × (RSD/*S*).

### 2.6. Isolation of Neutrophils

Fresh blood was collected in heparin-containing tubes from healthy human volunteers who fulfilled the following criteria: nonsmoker, fasted overnight and had not taking any medicine or supplements. PMNs were isolated from the blood by Ficol-gradient separation as described previously [14]. Using a haemocytometer and light microscope, cell suspensions were counted. Cell suspensions were diluted with HBSS to obtain a final cell suspension of 1 × 10^6^/mL. The use of human blood was approved by the Human Ethical Committee of Universiti Kebangsaan Malaysia (approval number FF/2012/Ibrahim/23-May/432-May 2012–August 2013). 

### 2.7. Cell Viability

 Cell viability was determined by the standard trypan blue exclusion method. The neutrophils (1 × 10^6^/mL) were incubated with 6.25 or 100 *μ*g/mL of plants extracts and 3.125 to 100 *μ*g/mL of pure compounds in triplicate at 37°C for 2 h. The blue dye uptake was an indication of cell death. The percentage viability was calculated from the total cell counts.

### 2.8. Chemiluminescence Assay

 Luminol-amplified chemiluminescence assays were carried out as described by Koko et al. [31]. Briefly, 25 *μ*L diluted whole blood or 25 *μ*L PMN (1 × 10^6^/mL) suspended in HBSS^++^ were incubated with 25 *μ*L of five different concentrations (6.25–100 *μ*g/mL). The cells were induced with 25 *μ*L of opsonized zymosan followed by 25 *μ*L of luminol as a probe, and then HBSS^++^ was added to adjust the final volume to 200 *μ*L. The final concentrations of the samples in the mixture were 12.5, 6.25, 3.13, 1.56, and 0.78 *μ*g/mL. Tests were performed in 96-well microplates which were incubated at 37°C for 50 min in the thermostated chamber of the luminometer. Luminol, 0.6% DMSO, HBSS^++^ and cells were added as a control, and the acetylsalicylic acid was used as a positive control. The final concentration of DMSO in the mixture was 0.6% to eliminate the effect of the solvent on the chemiluminescence. The luminometer results were monitored as chemiluminescence RLU (reading per luminometer unit) with peak and total integral values set with repeated scans at 30 s intervals and 1 s points measuring time. 

### 2.9. Chemotaxis Assay

 The assay was performed using a modified 48-well Boyden chamber with formyl-methionyl-leucyl-phenylalanine (fMLP) a bacterial peptide as a chemoattractant, as previously described by Sacerdote et al. [32]. Briefly, aliquots of 25 *μ*L of fMLP (10^−8^ M) were added to the lower chamber. Serial dilutions 5 *μ*L of each extract (6.25–100 *μ*g/mL) were added to the upper chamber containing PMNs (1 × 10^6^ cells per mL) suspended in HBSS^++^. The final concentrations of the samples in the mixture were 10, 5, 2.5, 1.25, and 0.625 *μ*g/mL. Then cells were incubated for 1 h at 37°C in a CO_2_ incubator. Migrated cells which had adhered to the distal part of the filters were fixed and stained by haematoxylin and xylene. The cell migration distance was measured by using a light microscope. Control wells contained chemoattractant buffer (DMSO and HBSS, 1 : 1 ratio). Ibuprofen was used as a positive control. 

### 2.10. Phagocytic Assay

The assay was carried out according to the protocol given by the manufacturer. Briefly, 100 *μ*L heparinized peripheral whole blood was incubated with 20 *μ*L FITC-labelled *E. coli* and 20 *μ*L of test samples (extracts: 6.25 and 100 *μ*g/mL; pure compounds: 3.125 and 50 *μ*g/mL) in a closed shake water bath at 37°C for 10 min, while the negative control was without test sample and remained on ice. Phagocytosis was quenched by adding ice-cold quenching solution to the mixture at the end of the incubation period. Then lysing solution was added for lysis of erythrocytes and fixation of the leucocytes. After a final wash the cells were resuspended in DNA staining solution to exclude aggregation artifacts of bacteria or cells and analyzed by flow cytometry. The phagocytic ability was evaluated in neutrophils and monocytes. Live populations were gated by the software program in the scatter diagram (FCS versus SSC). Phagocytic activity was determined as the percentage of phagocytizing neutrophils and monocytes.

### 2.11. Statistical Analysis

 All the data were analysed using Statistical Package for Social Sciences (SPSS) version 15.0. Each sample was measured in triplicate and the data presented as mean ± standard deviations (SD). The IC_50_ values were calculated using Graph Pad Prism 6 software. The values were obtained from at least three determinations. Data were analysed using a one-way analysis of variance (ANOVA) for multiple comparisons. *P* < 0.05 was considered to be statistically significant.

## 3. Results 

### 3.1. Isolation and Identification of Compounds

 In this study, phyllanthin and hypophyllanthin were isolated from the whole plants of *Phyllanthus amarus* from Malaysia and Indonesia by various chromatographic techniques. The compounds were identified by comparing their physicochemical and spectroscopic properties with literature values [33]. The marker compounds were obtained in high yields (phyllanthin, ranging from 1.15 to 2.29%; hypophyllanthin, ranging from 2.35 to 3.21%) from the crude methanol extracts of *P. amarus* from both countries. The isolated compounds were identified by their physicochemical properties, NMR and ESI-MS data, and compared with literature values [33].

### 3.2. Standardization of the Methanol Extracts of *Phyllanthus amarus* and *P. urinaria*

**Figure 1:**
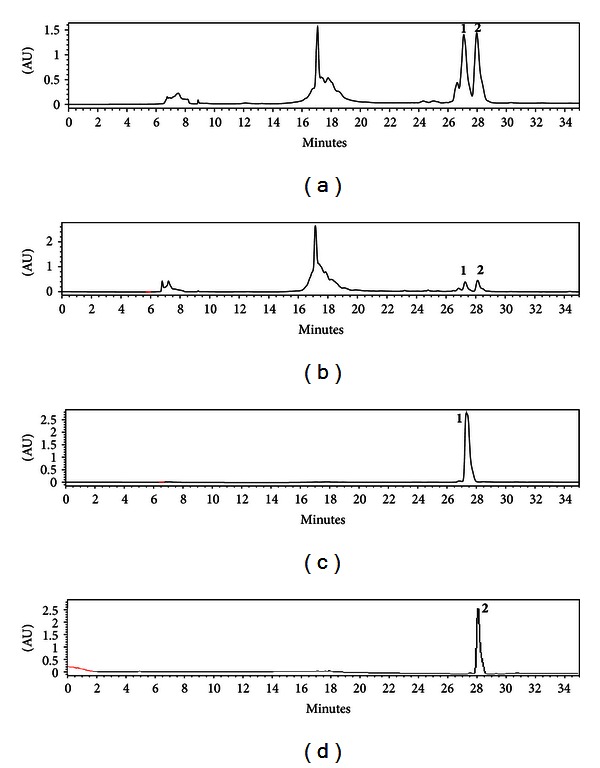
Representative HPLC chromatograms of (a) *Phyllanthus amarus* (Mal), (b) *P. urinaria* (Ind) standardized to phyllanthin (**1**) at RT = 27.251 min and hypophyllanthin (**2**) at RT = 28.079 min, (c) reference standard (phyllanthin) at 27.265 min, and (d) reference standard (hypophyllanthin) at 27.883 min.

 The chromatograms of the reversed-phase HPLC column of the methanol extracts of *P. amarus* and *P. urinaria* showed two major peaks of phyllanthin and hypophyllanthin, corresponding to retention times at 27.251 and 28.079 min, respectively. The peaks were identified by comparing them with HPLC of reference standards of phyllanthin and hypophyllanthin (Figure 1). Amounts of phyllanthin and hypophyllanthin (*μ*g/mL) in *Phyllanthus amarus* and *P. urinaria *obtained from HPLC measurements are shown in Table 1. Calibration curves plotted were linear over the concentration range of 31.25–250 *μ*g/mL with a correlation coefficient (*r* ^2^) of 0.9970 and 0.9973 for phyllanthin and hypophyllanthin, respectively. Reproducibility of the results was demonstrated where the % RSD values for interday assay precision of peak area and retention time were 1.6298 and 0.2162% for hypophyllanthin and 8.18 and 0.14% for phyllanthin, respectively, whereas the % RSD values for intra-assay precision of peak area and retention time were 6.1721 and 0.8589% for hypophyllanthin and 7.25 and 0.32% for phyllanthin, respectively. Limit of detection (LOD) and limit of quantification (LOQ) of hypophyllanthin were found to be 2.33 and 7.07 ng/mL, respectively, while for phyllanthin were found to be 5.48 and 16.6 ng/mL, respectively. 

### 3.3. Chemotaxis Assay

**Figure 2:**
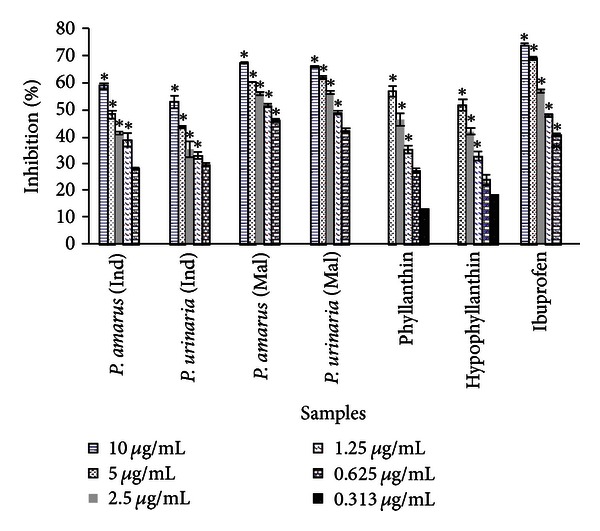
Percentage of inhibition of *Phyllanthus* sp. and their chemical markers on PMN chemotaxis. Data are mean ± SEM (*n* = 3). Significance of differences with respective control: *P < 0.05.

 Cells were viable (>95%) at 6.25 and 100 *μ*g/mL of the extracts after 2 h incubation. The highest concentration that cells were viable after 2 h incubation with phyllanthin and hypophyllanthin was 50 *μ*g/mL. The effects of the extracts and the isolates at the serial dilutions of 10 to 0.313 *μ*g/mL, on the migration of PMNs towards the chemoattractant (fMLP), were determined and their percentage inhibitions (%) are shown in Figure 2. All the extracts, particularly *P. amarus* and *P. urinaria* from Malaysia, showed strong inhibitory activity with a dose-dependent effect. Phyllanthin and hypophyllanthin also strongly inhibited migration of PMNs. Chemoattractant buffer (DMSO and HBSS, 1 : 1 ratio) was used as a control and ibuprofen was used as a positive control. All the samples showed a dose-dependent effect as shown in Figure 2. The IC_50_ values of the extracts and the compounds are shown in Table 3. 

### 3.4. Phagocytic Activity

**Figure 3:**
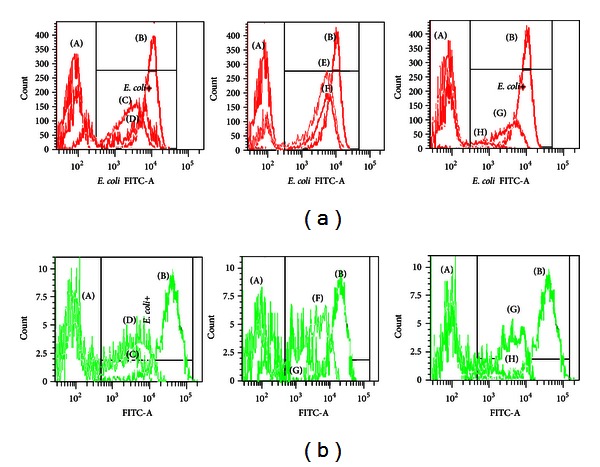
Representation of *E. coli* engulfment by neutrophils (3a) and monocytes (3b). (A) negative control, (B) positive control, (C) methanol extract of *P. amarus *(Mal) (D) methanol extract of *P. amarus *(Ind)(E) methanol extract of *P. urinaria *(Mal), (F) methanol extract of *P. urinaria, *(Ind), (G) hypophyllanthin (H) phyllanthin.

 The extracts from Malaysian *P. amarus* and *P. urinaria* at 100 and 6.25 *μ*g/mL showed moderate inhibition of bacteria engulfment by neutrophils and monocytes (Table 2 and Figure 3). Among the extracts, *P. amarus* (Malaysia) at 100 *μ*g/mL exhibited the highest engulfment inhibitory activity with percentage of phagocytizing cells of 69.2 and 50.8% for neutrophils and monocytes, respectively. The engulfment inhibitory activity at normal condition at 37°C was used as a positive control and normal condition at 0°C as negative control (Table 2). Phyllanthin at 50 *μ*g/mL exhibited strong inhibitory activity with percentage of phagocytizing cells of 14.2 and 27.1% for neutrophils and monocytes, respectively. 

### 3.5. Inhibition of Reactive Oxygen Species Generation

**Figure 4:**
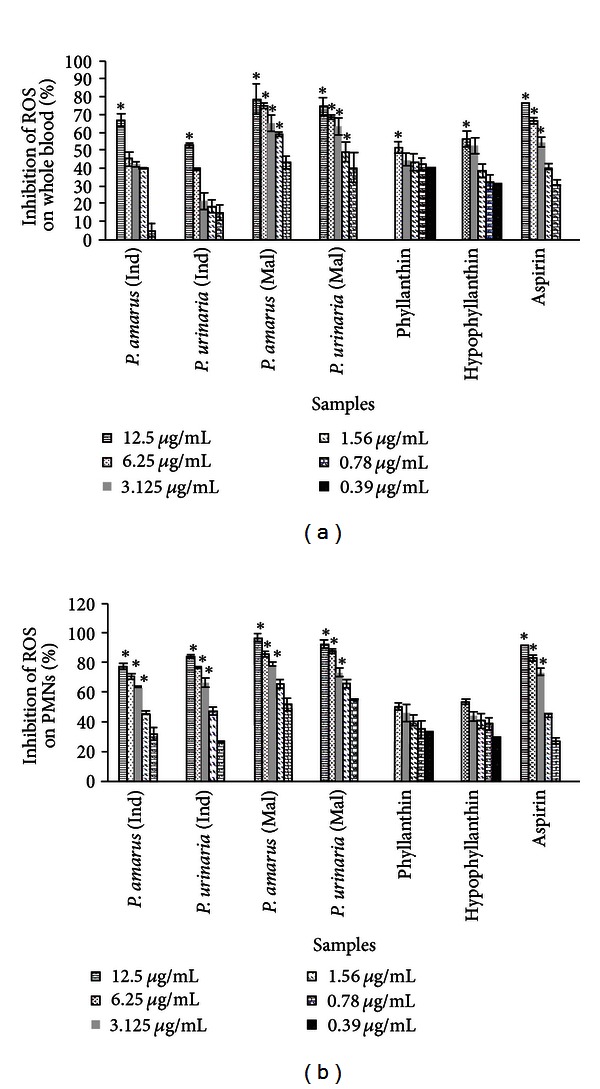
Percentage of inhibition of ROS inhibitory activity of *Phyllanthus* sp. and their chemical markers on whole blood (a) and PMNs (b) assayed by luminol amplified chemiluminescence. Data are mean ± SEM (*n* = 3). Significance of differences with respective control: *P < 0.05.

 Preliminary screening of the extracts of *P. amarus* and *P. urinaria* on the whole blood showed that *P. amarus* and *P. urinaria* from Malaysia exhibited high inhibitory activity for luminol-enhanced chemiluminescence with IC_50_ values of 1.1 and 1.6 *μ*g/mL, respectively (Table 3 and Figure 4). Phyllanthin and hypophyllanthin exhibited IC_50_ values of 8.8 and 8.4 *μ*M, respectively, indicating that they were more potent than the positive control, aspirin (12.2 *μ*M) (Table 3). The extracts were further investigated for their effects on the oxidative burst of PMNs. Of all the extracts, *P. amarus* and *P. urinaria* from Malaysia were the more potent samples against PMN with both extracts exhibited IC_50_ value of 0.7 *μ*g/mL which was much lower than that of aspirin (1.9 *μ*g/mL). All the extracts and isolates showed a dose-dependent effect as shown in Figure 4. Phyllanthin also showed lower IC_50_ value (7.6 *μ*M) than hypophyllanthin (14.2 *μ*M) and aspirin (10.5 *μ*M) on the oxidative burst of PMNs, indicating that it was a more potent inhibitor of ROS generation. 

## 4. Discussion

The extracts of *Phyllanthus* species were standardized for phyllanthin and hypophyllanthin contents by HPLC analysis. Quantitative determination of the marker compounds by HPLC indicated that *P. amarus* from Malaysia contained the highest amounts of phyllanthin (172.4 *μ*g/mL) and hypophyllanthin (214.1 *μ*g/mL) while *P. urinaria* from Indonesia contained the lowest concentrations of these marker compounds (Table 1). The variations in the quantitative amounts of the marker compounds in the plants of similar species collected from the two different locations are a response of the individual plant to environmental factors related to altitude or a genetic adaptation of the populations growing at different altitudes to specific environment [34].

 The cell viability test was performed using trypan blue to determine the nontoxic concentrations of *P. amarus* and *P. urinaria* extracts, and the isolated marker compounds (phyllanthin and hypophyllanthin). The high cell viability indicated that the extracts and the compounds were nontoxic to immune cells and could potentially modulate the cellular immune response in the reaction mixtures [14]. All the extracts strongly inhibited migration of PMNs with the extract of *P. amarus* from Malaysia showing the strongest inhibitory activity (IC_50_ value of 1.1 *μ*g/mL). Phyllanthin and hypophyllanthin showed relatively strong activity with IC_50_ values of 3.2 and 4.4 *μ*g/mL (7.6 and 10.2 *μ*M), respectively, which were slightly higher than the value for ibuprofen (1.4 *μ*g/mL or 6.8 *μ*M). Ibuprofen was found to be the most effective NSAIDs in blocking the migration of PMNs in a previous study [35]. 

 PMNs ability to phagocytize opsonized bacteria was evaluated by phagotest kit and analyzed by flow cytometry. The phagocytic activity was calculated by comparing the percentage of observed phagocytosis, by decrease in the percentage of *E. coli* ingestion by phagocytes. Complement-opsonized microbes are efficiently recognized by complement surface receptor on PMNs including CR1, CR2, and CR3, while antibody-coated microbes are recognized by neutrophils receptors specific for the Fc region of antibody, such as Fc*α*R, Fc*ε*RI, Fc*γ*RI, and Fc*γ*RII [14, 16]. The phagocytic activity of all the plant extracts and the marker compounds was less than the negative control, signifying that they were reducing the percentage of FITC-labelled *E. coli* ingestion and thus inhibiting the phagocytic cells (Table 2 and Figure 3). The results suggest that the moderate inhibitory of intake complement and immunoglobulin opsonized *E. coli* of the extracts might be due to the inhibition of those significant receptors. Phyllanthin showed the lowest percentage of phagocytizing cells, indicating the strongest inhibitory activity. The results suggest that phyllanthin might be the major contributor of the phagocytic activity of the plant extracts. 

 PMNs upon activation by serum-opsonized zymosan (SOZ) would accumulate at the site of inflammation and bind and destroy invading microorganisms by phagocytosis process. This mechanism triggers the generation of superoxide radicals and other secondarily derived ROS, such as hydrogen peroxide, hypochlorous acid, and cholarimines. The ROS were then quantified by the luminol-enhanced chemiluminescence assay. The luminol was used as probes to assess ROS production from plant extracts in PMN cells. The relatively small molecular weight of luminol enables it to enter the cells and subsequently to be oxidized by hydrogen peroxide to form a luminol radical, which eventually yields an unstable endoperoxide due to the interaction of luminol radical with other radicals [36]. This endoperoxide decomposes to an excited aminophthalate, which contributes to the emission of light [37]. In this study, all the extracts and isolates exhibited the inhibitory activity on ROS production by phagocytes in a concentration-dependent manner. Among the extracts, *P. amarus* and *P*. *urinaria* from Malaysia exhibited activity stronger than the positive control, aspirin. This inhibitory activity could be due to the ability of the extracts to block the interaction of SOZ with complement receptors, consequently inhibiting NADPH oxidase [36]. Phyllanthin and hypophyllanthin exhibited strong inhibitory activity on the oxidative burst in whole blood with IC_50_ values (8.8 and 8.4 *μ*M, resp.) lower than that of aspirin (12.2 *μ*M). In agreement with the inhibition on phagocytic activity, phyllanthin also showed a lower IC_50_ value (7.6 *μ*M) than hypophyllanthin and aspirin on the oxidative burst of PMNs, indicating that it was a more potent inhibitor of ROS generation. Aspirin was used as a positive control based on a previous report that the drug inhibited luminol-amplified chemiluminescence of human neutrophils [38]. 

## 5. Conclusion 

The standardized methanol extracts of *P. amarus* and *P. urinaria* and their biomarkers phyllanthin and hypophyllanthin were able to modulate the innate immune response of phagocytes especially on the chemotactic migration of phagocytes, phagocytic ability, and on the release of ROS. Among the extracts, the Malaysian *P. amarus* consistently showed strong inhibition at different steps of the phagocytosis, emphasizing its potential to be developed into a standardized immunomodulating product. Phyllanthin exhibited higher inhibitory effects on the phagocytic activity of neutrophils particularly in inhibiting ROS production and bacteria engulfment as compared to hypophyllanthin. The high inhibitory activity of the extracts could be due to the high amounts of phyllanthin and hypophyllanthin present although the synergistic effect of the other constituents of the plant extracts should not be excluded. *P. amarus* and *P. urinaria* and their biomarkers have potential to be sources of leads for development of new immunomodulatory agents. However, further studies are required to elucidate their activities on other mechanisms of immunomodulatory responses.

## Figures and Tables

**Table 1:** Amounts of phyllanthin and hypophyllanthin (*μ*g/mL) in *Phyllanthus amarus* and *P. urinaria *obtained from HPLC measurements.

Species	Phyllanthin	Hypophyllanthin
*P. amarus *(Mal)	172.4	214.1
*P. amarus *(Ind)	93.3	124.6
*P. urinaria *(Mal)	96.8	111.2
*P. urinaria *(Ind)	9.0	14.8

**Table 2:** Percentage of phagocytic activity (%) of neutrophils and monocytes at various concentrations of *Phyllanthus* extracts and their chemical markers (Mean ± SEM, *n* = 3).

Sample (*μ*g/mL)	Neutrophils				Monocytes			
	100	50	6.25	3.125	100	50	6.25	3.125
*P. amarus* (Ind)	89.2 ± 3.2		89.8 ± 6.2		70.8 ± 8.2		71.0 ± 4.1	
*P. urinaria* (Ind)	85.1 ± 2.4		86.5 ± 7.5		52.3 ± 9.8		86.4 ± 4.5	
*P. amarus* (Mal)	69.2 ± 3.6		81.7 ± 2.9		50.8 ± 2.3		55.5 ± 1.6	
*P. urinaria* (Mal)	73.7 ± 6.2		74 ± 4.0		52.3 ± 6.6		60.1 ± 5.0	
Hypophyllanthin		49.1 ± 4		80.5 ± 3.8		64.6 ± 10.8		67.0 ± 3.0
Phyllanthin		14.2 ± 2		34.9 ± 4.2		27.1 ± 5.7		52.1 ± 2.6

**Table 3:** IC_50_ values (*μ*g/mL) of ROS inhibitory and chemotaxis activities of *Phyllanthu*s sp. and their chemical markers on phagocytes (Mean ± SEM, *n* = 3). IC_50_ values in *μ*M are in parentheses.

Sample	Chemotaxis	Chemiluminescence	
		Whole blood	PMNs
*Phyllanthus amarus* (Ind)	5.1 ± 0.4	5.2 ± 1.1	1.9 ± 0.1
*Phyllanthus urinaria* (Ind)	5.4 ± 0.7	11.9 ± 0.9	1.9 ± 0.1
*Phyllanthus amarus* (Mal)	1.1 ± 0.1	1.1 ± 0.9	0.7 ± 0.2
*Phyllanthus urinaria* (Mal)	1.4 ± 0.2	1.6 ± 0.5	0.7 ± 0.3
Phyllanthin	3.2 ± 0.1	3.7 ± 2.8	3.2 ± 0.8
	(7.6 ± 0.1 *μ*M)	(8.8 ± 1.0 *μ*M)	(7.6 ± 0.4 *μ*M)
Hypophyllanthin	4.4 ± 0.2	3.6 ± 1.4	6.1 ± 2.3
	(10.2 ± 0.4 *μ*M)	(8.4 ± 1.3 *μ*M)	(14.2 ± 1.7 *μ*M)
Ibuprofen	1.4 ± 0.1		
	(6.8 ± 0.1 *μ*M)		
Aspirin		2.2 ± 0.8	1.9 ± 0.2
		(12.2 ± 1.1 *μ*M)	(10.5 ± 2.1 *μ*M)
